# Micronutrient supplementation for cancer prevention

**DOI:** 10.1186/1897-4287-13-S2-A1

**Published:** 2015-11-26

**Authors:** Alan Kristal

**Affiliations:** 1 Cancer Prevention Program, Fred Hutchinson Cancer Research Center, Seattle, WA, USA

## 

Decades of research on the use of micronutrient supplements for cancer prevention have yielded inconsistent and disappointing results. Here I reflect upon the limitations of past research, and develop a framework for how to move from basic biological and epidemiological research to large, randomized cancer prevention trials. There are many biologically rational hypotheses supporting effects of micronutrient supplements for cancer prevention, with thousands of supportive studies in *in vitro *and animal models. But careful evaluation of much of this research shows it irrelevant to human nutrition: agents tested are in concentrations and forms that are never seen *in vivo*. Small clinical studies often have no control groups, use uninformative endpoints, or have obvious design or analysis flaws. Epidemiological studies are challenging because they can rarely capture biological complexity: associations may differ by genetic characteristics or environmental exposures (e.g., smoking), and cancers with diverse phenotypes are grouped by anatomic site. Furthermore, because supplement use is associated with many other health-related behaviors and most supplement users take multiple supplements, statistical methods cannot reliably disentangle these highly inter-correlated factors. Studies based on blood-based biomarkers of micronutrient intake can be misleading, either because the biomarker is an acute phase reactant (Se, α-tocopherol) or the biomarker is not a valid measure of micronutrient status (Zn). Randomized clinical trials have a high likelihood of yielding null results, because often the optimal dose and formulation of the agent are unknown, adherence is poor, study duration is too short, or the outcome is misspecified. Furthermore, many studies have found that micronutrient supplementation increases cancer risk, suggesting the high dose micronutrient supplementation can lead to subacute toxicity. The challenge to biomedical research is to use *in vitro *and animal experimental models that are relevant to human nutrition, design and execute small clinical studies with the same rigor required for pharmaceutical research, and use micronutrient biomarkers that validly reflect nutritional status. Clinical trials should more ahead only when the biology is well-understood, the population likely to benefit can be identified, and the funding is sufficient to support programs to promote treatment adherence and the collection and banking of biological specimens at randomization and at multiple times post-randomization.

